# Pulmonary Kaposi sarcoma in a human immunodeficiency virus – infected woman: a case report

**DOI:** 10.1186/1757-1626-2-5

**Published:** 2009-01-02

**Authors:** Rafael Ferracini Cabral, Edson Marchiori, Tatiana Chinem Takayasu, Fernanda Caseira Cabral, Raquel Ribeiro Batista, Gláucia Zanetti

**Affiliations:** 1 Service of Diagnostic Radiology, Clementino Fraga Filho Universitary Hospital, Rio de Janeiro, Brazil; 2 Department of Radiology, Faculty of Medicine, Federal University of Rio de Janeiro, Rio de Janeiro, Brazil

## Abstract

Approximately 90% to 95% of Kaposi sarcoma cases occur in human immunodeficiency virus – infected homosexual and bisexual men. Pulmonary Kaposi sarcoma is uncommon in women, and rarely considered as a potential cause of diffuse lung disease in women with acquired immunodeficiency syndrome. The disease is usually mistaken clinically for pulmonary infection. A 32-year-old woman was admitted with a 2-month history of dyspnea, evening fever, hemoptysis, weight loss, and generalized adenomegaly. Physical examination showed erythematous macules in the lower limbs. Skin and open lung biopsy demonstrated Kaposi sarcoma. Computerized tomography demonstrated peribronchovascular interstitial thickening. Although uncommon, pulmonary Kaposi sarcoma should be considered in the differential diagnosis of diffuse lung disease in women with AIDS.

## Introduction

Kaposi sarcoma (KS) is a low-grade mesenchymal tumor involving the blood and lymphatic vessels that primarily affects the skin and causes disseminated disease in a variety of organs. Acquired immunodeficiency syndrome (AIDS)-associated KS predominantly affects homosexual and bisexual men. The intrathoracic KS literature consists of several series of men [1] and rarely includes cases in women [2]. Accordingly, there has been a low index of suspicion for diagnosing KS in women with AIDS. In those patients who are not severely immunocompromised, KS may remain an indolent cutaneous disease. However, in advanced AIDS, KS will often disseminate to involve the oropharynx, larynx, tracheobronchial tree, lungs, and other viscera [3].

## Case presentation

A 32-year-old woman was admitted one year after being diagnosed with lymph node tuberculosis and AIDS. She had received treatment with rifampicin, hydrazine, and pyrazinamide for 6 months, as well as zidovudine and didanosine, which were used irregularly. She presented with a 2-month history of evening fever, progressive dyspnea, cough, hemoptysis, weight loss (4 kg in 2 months), and generalized adenomegaly.

At admission, her weight was 53 kg and and her height was 1.70 m. Physical examination showed the patient to be ill-looking, emaciated, pale, acyanotic and afebrile. Her blood pressure was 90/60 mm Hg, her pulse rate was 110 beats per minute, and her respiratory rate was 36 breaths per minute. Cervical, submandibular, and axillary lymphadenopathy were noted. There was crackles in the middle third of the lungs bilaterally. Multiple erythematous macules were observed on the lower limbs.

Laboratory tests showed the following: erythrocytes 2,900/mm^3^, hemoglobin 8.5 g/dL, hematocrit 29.1%, leukocytes 5,570/mm^3 ^(band neutrophils 5%, segmented neutrophils 64%, eosinophils 3%, lymphocytes 10%), platelets 138,000/mm^3^. On 3 L/min supplemental oxygen performed by nasopharyngeal catheter, the patient's blood pH was 7.40, her arterial oxygen tension (PaO_2_) was 52.3 mm Hg, arterial carbon dioxide tension (PaCO_2_) 33.8 mm Hg, and peripheral oxygen saturation (SaO_2_) 89.3%. Additional laboratory tests revealed blood urea nitrogen at 18 mg/dL, creatinine at 0.5 mg/dL, CD4 at 252 cells/mm^3 ^(18%), and CD8 at 812 cells/mm^3 ^(58%). Ziehl Neelsen's stain for acid-fast bacilli of the sputum was negative.

**Figure 1:**
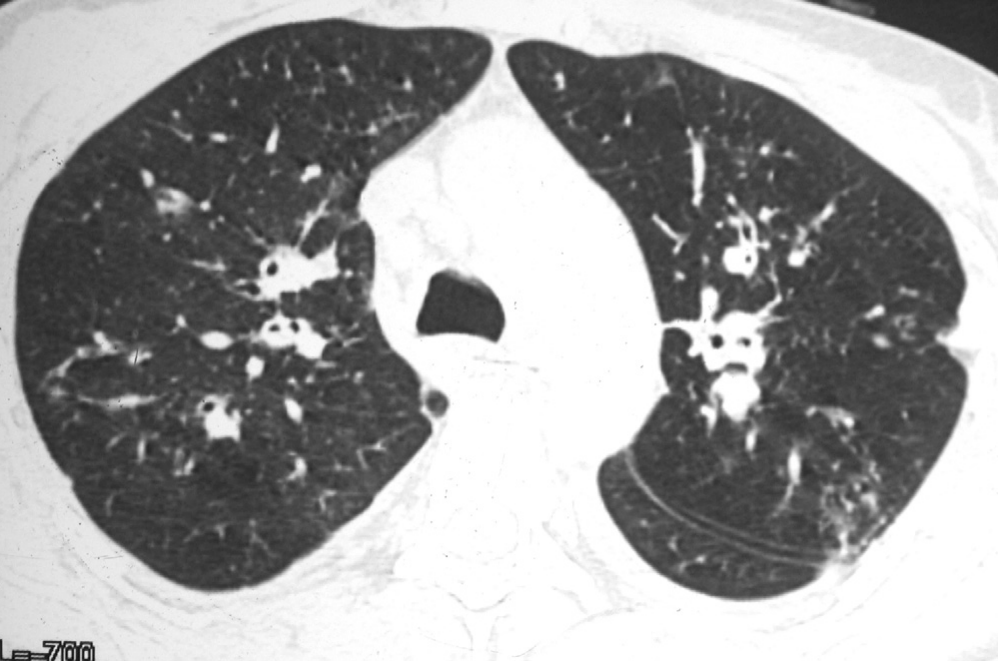
**HRCT scan obtained with lung window shows diffuse peribronchovascular thickening and small nodules bilaterally**. There are also nodules in the fissures and interlobular septal thickening.

**Figure 2:**
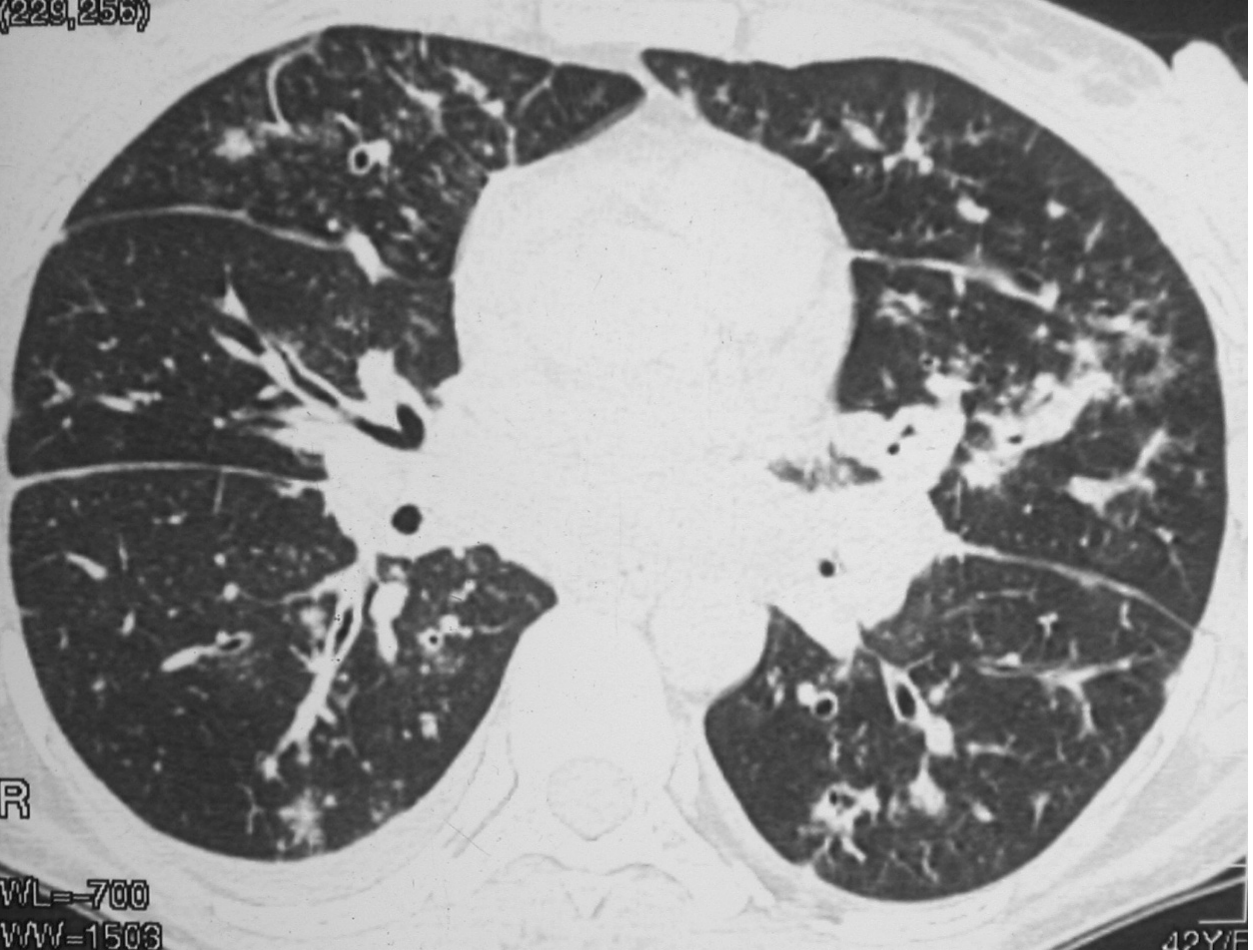
**HRCT scan obtained with lung window shows diffuse peribronchovascular thickening and small nodules bilaterally**. There are also nodules in the fissures and interlobular septal thickening.

**Figure 3:**
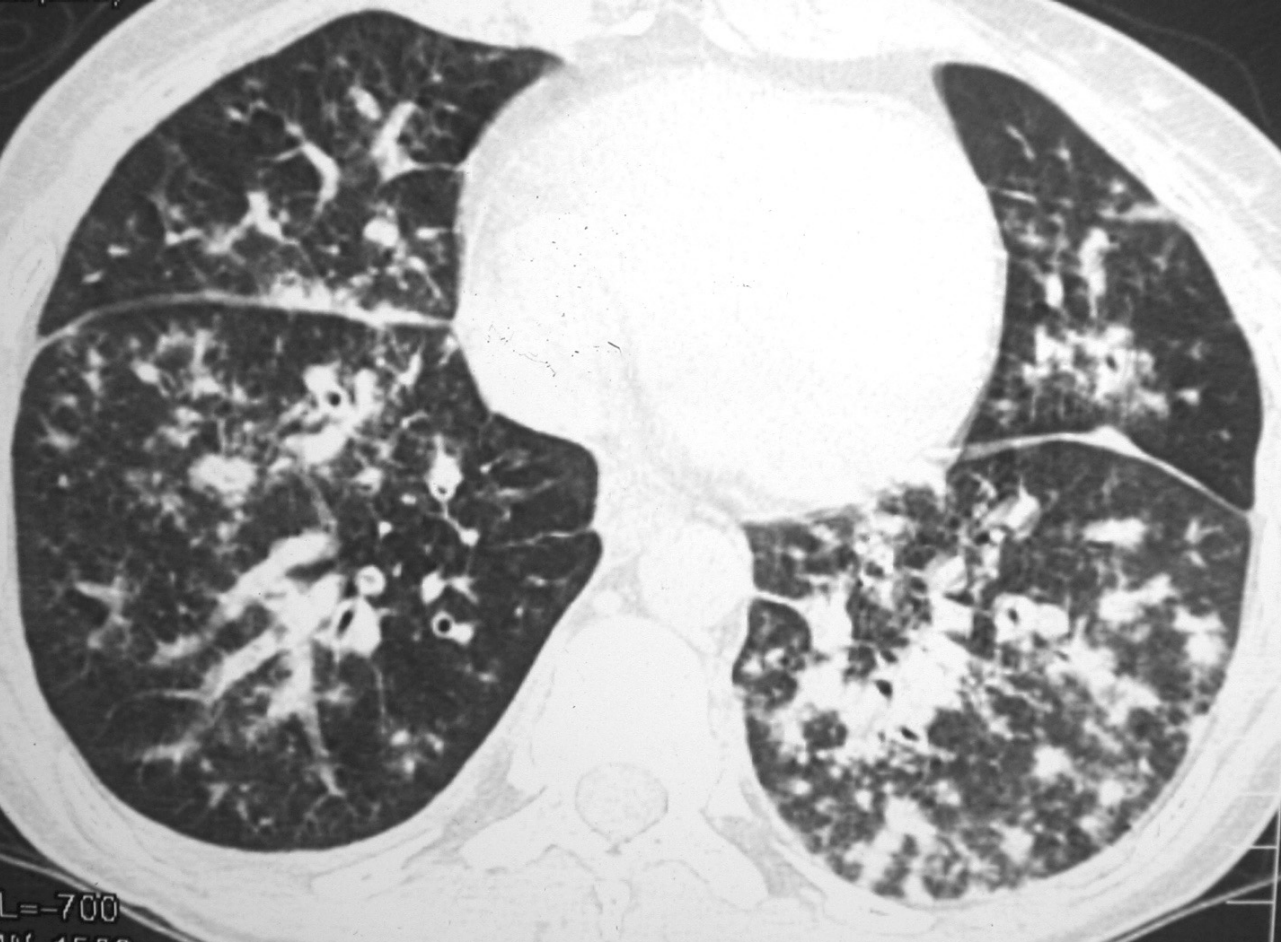
**HRCT scan obtained with lung window shows diffuse peribronchovascular thickening and small nodules bilaterally**. There are also nodules in the fissures and interlobular septal thickening.

The chest radiograph revealed mild interstitial infiltration predominantly in perihilar regions. High-resolution computed tomography (CT) showed peribronchovascular interstitial thickening, with small parenchymatous nodules and discrete thickening of interlobular septa. There was also a small right pleural effusion (Figures 1, 2 and 3). Skin biopsy of a lesion on the left leg showed KS. Open lung biopsy was performed and showed a bronchial involvement by KS. Staining for fungi and mycobacteria were negative.

When results of the biopsy were received, the patient was started on chemotherapy with vincristine 2 mg, doxorubicin 30 mg, and bleomycin 15 mg once daily. The patient responded well to treatment, with improvement in her systemic symptoms and regression of the pulmonary lesions. One year after the end of chemotherapy, the patient is asymptomatic.

## Discussion

Thoracic disease is found in approximately 45% of patients with cutaneous AIDS-related KS [4,5]. Manifestations include parenchymal, tracheal, lymphatic, pleural, and chest wall abnormalities. Common clinical symptoms are chronic cough, dyspnea, fever, and hemoptysis [6]. A typical purplish endoscopic appearance of tracheobronchial KS is helpful in developing the differential diagnosis. About 20% of deaths are related to complications of the disease itself (upper airway obstruction, hemorrhage, or parenchymal destruction), but the majority of deaths are related to other factors (e.g., concomitant infection) [7]. A low CD4 lymphocyte count (100 cells/mm^3^) generally accompanies thoracic involvement by AIDS-related KS [8]. In the present case, the last CD4 before these symptoms was 252 cells/mm^3^.

Radiographically, AIDS-related KS may manifest as an isolated finding or in association with an opportunistic infection. In the first scenario, radiography may demonstrate middle to lower lung zone reticular opacities and parenchymal nodules. Some KS patients may present with normal chest radiographic findings [4].

The characteristic high-resolution CT manifestations of KS consist of peribronchovascular interstitial thickening and irregular or ill-defined nodules in a predominantly peribronchovascular distribution. These findings reflect the propensity of KS cells to infiltrate the perihilar peribronchovascular interstitium [2]. Other common findings include thickening of the interlobular septa, lymphadenopathy, and pleural effusion. Interlobular septal thickening can result from infiltration by tumor cells or edema [9].

In this report, we present a case of pulmonary KS in a woman with AIDS. The majority of women with similar clinical problems were initially suspected of having an infectious etiology for diffuse pulmonary disease [3]. Performing a careful skin examination is particularly important in these cases. Epidemiologic evidence has suggested a sexually transmitted infectious etiology for KS for some time, and human herpesvirus 8 (HHV-8) has been proposed as the etiologic infection [10]. HHV-8 induced inflammation, angiogenesis, and oncogenesis are critical for the development of KS [11]. Although KS remains a disease that is seen predominantly in men, heightened awareness of the occurrence of this disease in women may lead to its diagnosis and treatment at an earlier stage.

## Abbreviations

KS: Kaposi's sarcoma; AIDS: acquired immunodeficiency syndrome; HIV: human immunodeficiency virus; HHV-8: herpesvirus 8

## Competing interests

The authors declare that they have no competing interests.

## Authors' contributions

RFC conceived the study. TCT, FCC and RRB performed the literature review. RFC, GZ and EM edit and coordinated the manuscript. All authors read and approved the final manuscript.

## Consent

Written informed consent was obtained from the patient for publication of this case report and accompanying images. A copy of the written consent is available for review by the Editor-in-Chief of this journal.
